# Experiences with an Inquiry-Based Ionic Liquid Module in an Undergraduate Physical Chemistry Laboratory

**DOI:** 10.1021/acs.jchemed.3c00871

**Published:** 2024-04-05

**Authors:** Kevin E. Riley, Samrat Dutta

**Affiliations:** Department of Chemistry, Xavier University of Louisiana, New Orleans, Louisiana 78125, United States

**Keywords:** Upper Division Undergraduates, Ionic Liquids, Physical Chemistry, Inquiry-Based/Discovery Learning

## Abstract

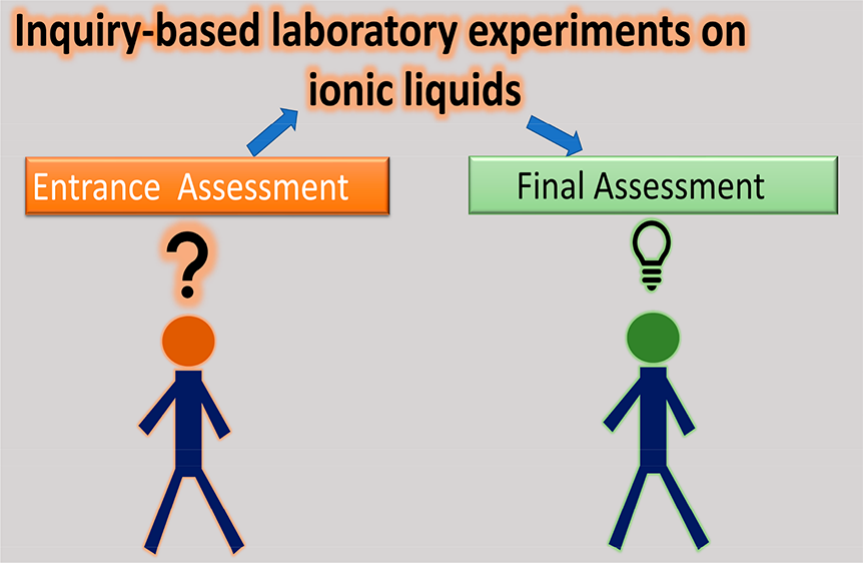

The topic of ionic liquids is typically not taught at the undergraduate level. Many properties, such as conductivity, vapor pressure, and viscosity, of these so-called “green solvents” are unique compared to traditional molecular solvents. Using active learning techniques, we introduced an ionic liquid module in the physical chemistry laboratory where their structures and physical properties, namely, viscosity, conductivity, and vapor pressure, were explored in relation to molecular solvents. Summative and formative assessments show that a majority of the participants were able to grasp the key concepts of ionic liquids. We envision that our methods and strategies can be one of the building blocks of introducing ionic liquids into the undergraduate chemistry curriculum.

Bringing green chemistry to undergraduate classrooms, particularly in minority institutions, is necessary to prepare students for the future and diversify the workforce. In recent years, there has been increased attention within academia and industry on ionic liquids due to their promising environmentally friendly applications.^1−3^ Considering that ionic liquids are relatively new in usage compared to common solvents, most undergraduates are unlikely to be exposed to them in their academic careers. In this manuscript, we provide our experiences in introducing student-oriented laboratory methods on ionic liquids in an upper-division undergraduate physical chemistry laboratory at our historically black university, Xavier University of Louisiana. We envision that the approaches presented here can be universally applied in undergraduate laboratory modules, providing a framework for familiarizing students with ionic liquid-based technologies, such as energy storage devices, carbon storage and sequestration, biomass pretreatment, lubricants, and heat transfer devices.

Ionic liquids are nonaqueous fluids that are liquid at or below room temperature. These liquids are entirely made of ions and typically consist of an organic cation and an organic or inorganic anion (Figure 1). Such liquids have many benefits over traditional molecular organic solvents, including but not limited to negligible vapor pressure, high thermal stability, high conductivity, nonflammability, and a large electrochemical window.^4,5^ Many of these physical and chemical properties can be tuned by simply changing the combination of cations and anions, opening many new applications of these liquids, including liquid–liquid extraction, wet-chemical synthesis, catalysis, electrochemical processes, extraction of metals, gas separation, biomass processing, pharmaceuticals, tribology, and electrolytes in energy storage devices.^6,7^ As these liquids are made of ions, their properties are governed by dominant Coulomb forces in subtle balance with other interactions such as hydrogen bonding and dispersion forces.^3^

**Figure 1:**
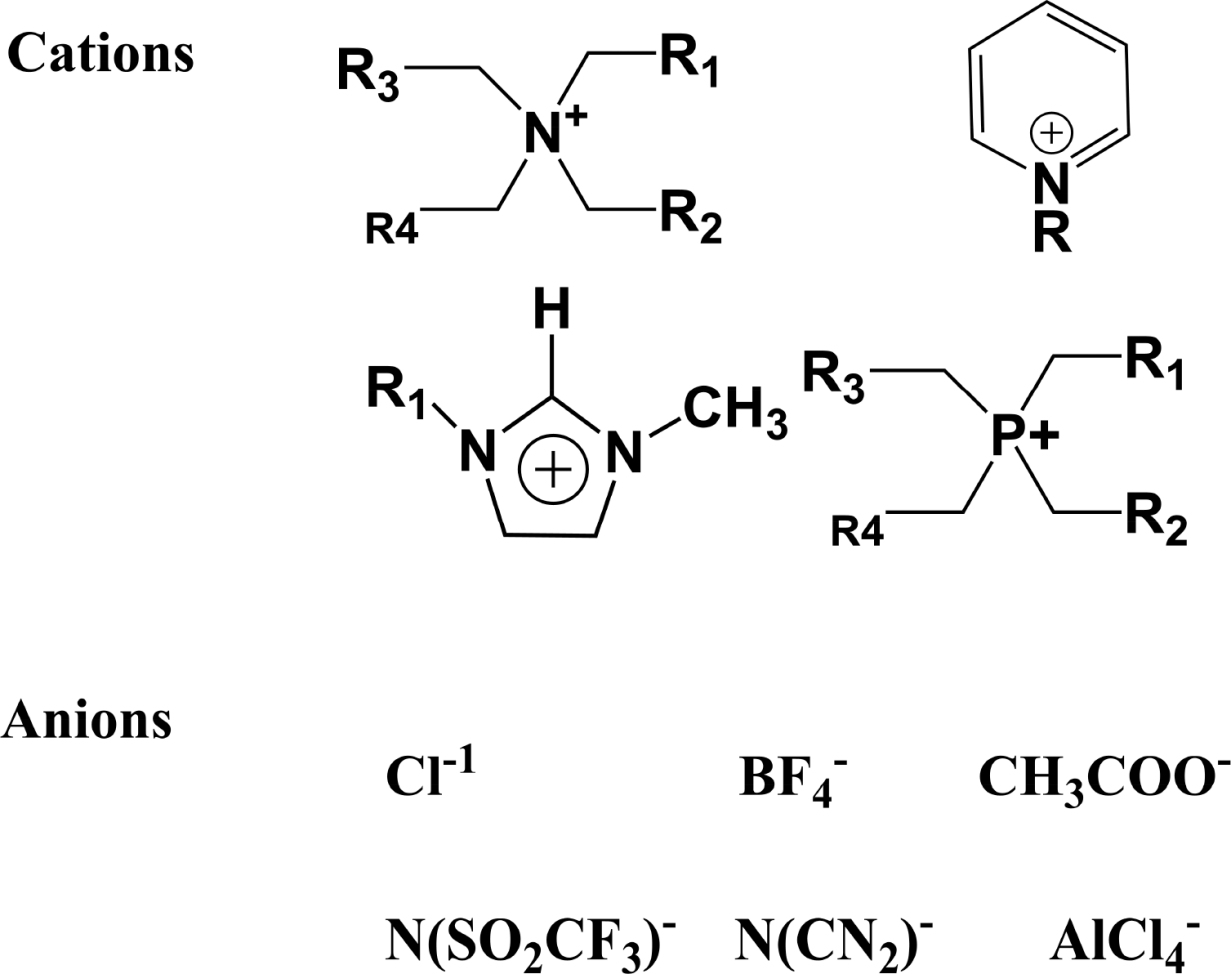
Common cations and anions which can form ionic liquids.

Teaching undergraduate students the properties of ionic liquids can be challenging, as many properties of these liquids do not follow general trends observed for common organic solvents. For example, the vapor pressure of organic liquids obeys the Clausius–Clapeyron equation, but ionic liquids have negligible vapor pressure under normal conditions; so, a relationship between vapor pressure and temperature does not arise for these liquids.^8^ The problem is similar to teaching classical and quantum mechanics. Many students see quantum mechanics as counterintuitive because it is removed from classical everyday experience.^9^ The same applies to ionic liquids in the context of student experience with solvents which is either water or common organic solvents. Two common prior knowledge conditions can impede student learning^10^ on ionic liquid properties: (a) insufficient relevant background knowledge on ionic liquids and (b) inert prior knowledge in which students possess relevant prior knowledge but have difficulty transferring knowledge to new material. Inert prior knowledge, which includes but is not limited to Coulombic forces, hydrogen bonding, van der Waals forces, crystal packing, and heterogeneity,^11,12^ is relevant to interpret the molecular origins of ionic liquid properties. However, students can find it difficult to connect these concepts to ionic liquid characteristics.

Inquiry-based laboratory design, particularly those inspired by Process Oriented Guided Inquiry Learning (POGIL), provides a template to teach the complex topic of ionic liquids.^13−15^ POGIL is a structured inquiry-based teaching method where the instructor acts as a facilitator working with a small group of students on specially designed activities that guide the students through the three phases of the learning cycle: exploration, concept invention, and application. Such guided investigation generates useful and applicable knowledge, yields better results, improves understanding, and enhances the motivation of students to learn science.^16^ Though POGIL was designed to replace lectures in the classroom, Vishnumolakala et al.^17^ suggest that POGIL can be modified in accordance with the institution’s learning environment. Reports on POGIL and modified POGIL in laboratory settings show that blending teaching methods depending on the requirements can improve learning outcomes.^18−20^ Walker et al.^21^ suggest that a cautious balance of student-centered activities and traditional-style instruction may be the best approach to teaching. Hybridizing different pedagogies with the intent of optimizing the beneficial elements of each is proven to facilitate student understanding.^22,23^ A recent critical review^24^ concludes that a combination of inquiry-based and direct instruction is possibly the most effective way of teaching. Instructors can use an inquiry-based lesson with a traditional class discussion to present new information or clarify the underlying principles of the laboratory explorations.^25^

The POGIL workflow reported for physical chemistry laboratory (PCL) courses, the POGIL-PCL model,^26,27^ along with other related POGIL activities^23,28^ in the physical chemistry laboratory provide pathways for designing inquiry-based laboratory modules on ionic liquids. However, executing the POGIL workflow as reported in the literature in the laboratory for ionic liquids is difficult for two reasons: (a) ionic liquid is not taught as a topic in the chemistry curriculum at Xavier University of Louisiana, which we assume is true for many other universities and (b) the characteristics of these liquids are not similar to traditional solvents which the students are accustomed to in their curriculum, particularly in laboratory practices. In other words, students do not have the content knowledge or experience in handling ionic liquids. Our approach was to introduce imidazolium-based ionic liquids in the physical chemistry laboratory by blending elements of POGIL and traditional teaching methods, keeping in view students’ academic experience in this HBCU. The broad goal was to give students hands-on experience handling ionic liquids and learn their properties through self-discovery. The summative assessment shows that more than 80% of the students were able to gain new practical knowledge on ionic liquids, including melting point characteristics, viscosity, electrical conductivity, and vapor pressure.

## Academic Setting and Learning Goals

The module was designed to give students their first experiences in ionic liquids through an upper-division PCL course in the undergraduate chemistry curriculum. Students enrolled in the class had previously completed quantitative analysis and general physics courses and were currently enrolled or had earlier taken introductory physical chemistry courses. So, students know the fundamental concepts regarding intermolecular forces and their relationship to macroscopic properties, such as viscosity, electrical conductivity, and vapor pressure of molecular liquids but not for ionic liquids. The module was designed to facilitate novice-level knowledge and skills knowledge in ionic liquids for the students.

After completing this module, students are expected to

(a) Be conversant with ionic liquids, particularly with imidazolium-based ionic liquids, and be able to handle these liquids safely.
(b) Be familiar with the physical properties of ionic liquids.
(c) Be able to compare and contrast the physical properties of ionic liquids with conventional solvents.
(d) Have a novice-level understanding of the complex and often abstract concepts related to the origins of macroscopic properties of ionic liquids.

It is important to note that the experiments are designed to represent the general trend in the investigated property in imidazolium-based ionic liquids as a class of solvents. It does not mean that molecular solvents cannot have similar properties.

## Laboratory Design

### Entry and Exit Tickets

A total of 37 students spanning over three semesters were randomly selected based on the criteria that both entry and exit assessments are available. The entry assessment was done at the beginning of the laboratory module, whereas the exit assessment was inserted in the final examination of the course. The entry ticket questionnaire was developed in line with the concept inventory but keeping in view the students’ experience and curriculum at the Xavier University of Louisiana. Four multiple-choice questions were presented at the beginning of the class. A brief statement about ionic liquids preceded each of the questions (See Supporting Information, S1) to activate prior knowledge of their properties. The questions were designed to assess students’ (a) awareness of the existence of ionic liquids, (b) knowledge of the vapor pressure of ionic liquids, (c) knowledge of the conductivity of the ionic liquids, and (d) knowledge of the viscosity of ionic liquids.

As with the entry ticket, four multiple-choice questions were asked in the exit assessment. The questions were designed to probe students’ deeper understanding of ionic liquid properties (See Supporting Information, S2). Briefly, students were asked to indicate if (a) imidazolium-based ionic liquids have high, low, or negligible vapor pressure, (b) the conductivity of ionic liquid could be best described as a nonelectrolyte, weak electrolyte, or strong electrolyte, (c) the viscosity of these liquids was greater or lower than common solvents, and (d) the origins of the low melting point of ionic liquids was due to strong crystal packing, weak crystal packing, asymmetricity of ions, or symmetricity of ions.

### Inquiry-Based Laboratory Module

The 3 h physical chemistry laboratory session was divided into two parts: The first part aimed to build students’ content knowledge of ionic liquids, and the second part consisted of guided experimental explorations of selected properties of ionic liquids. The activities were done in groups of two to four students. The first and the second part had exploration and application cycles that were similar to POGIL.

The first part of the laboratory exercise involved a traditional lecture on the relationship between cation/anion size and melting point, with one of the main focuses being on how ionic liquids are generally composed of large molecular ions. The lecture was followed by a guided classroom activity. In this guided workflow, a handout was given in the exploration cycle to compare and contrast ionic liquid structures with common ionic compounds with instructor-guided discussions. In the application cycle, the students were asked to reflect on the possible reasons for the low melting point of ionic liquids using models as prompts provided by the instructor.

**Scheme 1:**
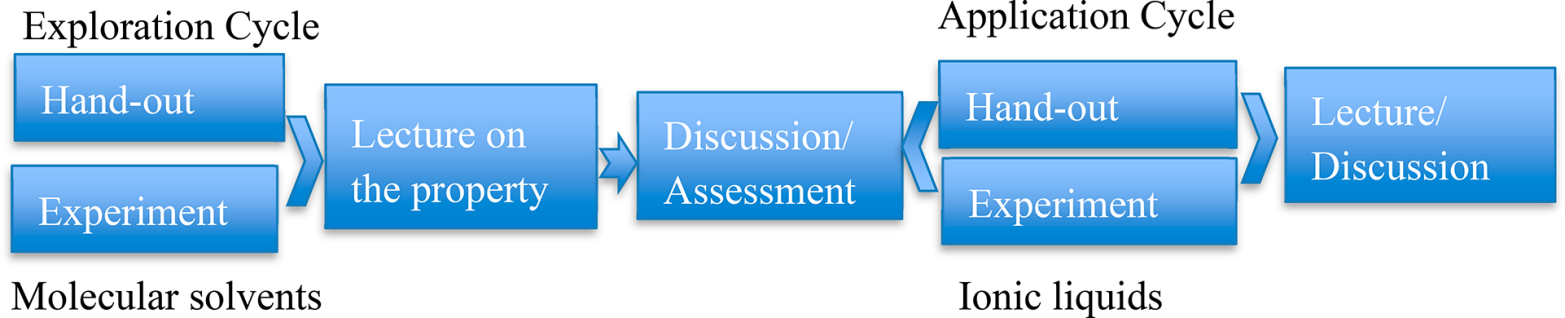
Inquiry-Based Experimental Design on Ionic Liquids

The second part of the laboratory module involved three experiments, namely, viscosity, conductivity, and vapor pressure, using an inquiry-based structure inspired by the POGIL workflow (Scheme 1). Viscosity, vapor pressure, and conductivity were chosen for exploration as students have prior knowledge, both theoretical and practical, of these properties from previous courses or earlier modules within this laboratory course. Typically, the POGIL workflow involves exploration, invention, and application cycles. However, instead of invention, a short, traditional lecture was inserted into the workflow to build on the concepts introduced in the first part of the laboratory session. The design of the exploration cycle in each of the three experiments consists of a handout and a guided experimental investigation of a given property of molecular solvents. The handout briefly describes the property and procedure for the experiment. As different groups may be given different liquids, the instructor facilitates sharing data with all learners so that they have all the information. After the students recollected their prior knowledge and assessed the collected data, the instructor delivered a short lecture relevant to that specific property of ionic liquid, culminating with open-ended questions such as “Based on the lecture and exploration, what do you think will be the trend of that property on ionic liquid?” As each group discussed the possible trend, the instructor prompted the students to think critically, particularly on the dominance of Columbic forces. A verbal assessment followed the discussion where a student from each group summarized the group’s view on the topic. The students were then asked to explore the same property on ionic liquids in the application cycle without revealing the answer. The exact details of the experiments in the application cycle were not necessarily the same as the exploration cycle. A postexperiment lecture and/or discussion followed each application cycle to reflect on the molecular origins of the ionic liquids’ physicochemical properties. This instructor-guided discussion/lecture is important as there can be nonintuitive and complex origins and trends of macroscopic properties in ionic liquids.

## Experimental Methods

1-Ethyl-3-methylimidazolium tetrafluoroborate ([EMIM][BF_4_]), 1-ethyl-3-methylimidazolium bis(trifluoromethylsulfonyl)imide, 1-ethyl-3-methylimidazolium ethyl sulfate ([EMIM][EtSO_4_]), 1-ethyl-3-methylimidazolium triflate ([EMIM][CF_3_SO_3_]), 1-ethyl-3-methylimidazolium acetate ([EMIM][CH_3_COO]), and 1-ethyl-3-methyl imidazolium tris(pentafluoroethyl)trifluorophosphate ([EMIM][FAP]) were used. All ionic liquids and common solvents were either obtained from Fisher Scientific or Sigma-Aldrich. All ionic liquids were dried at a high vacuum at 80 °C before the start of the class. The measurements taken in the class for an investigated ionic liquid property were qualitative, not quantitative. The workflow of experiments is designed to have minimum waste and maximum recovery of ionic liquid materials. More details are given in the instructor guide section on the experimental setup in the Supporting Information.

### Viscosity Measurement

The students used the viscosity race experiment in the exploration cycle to compare the viscosity of ionic liquids with common solvents. Briefly, a wooden panel was covered with fluorinated ethylene propylene (FEP) film and placed at an angle of 45°. Students were asked to note the time required for a given liquid to flow from the start point to the end point of the film. The students used water, DMSO, benzene, acetone, motor oil, and glycerol, along with the ionic liquids mentioned above, for the viscosity race experiment. In the application cycle, students used a Cannon-Fenske Routine viscometer to calculate the dynamic viscosity of the ionic liquids, [EMIM][EtSO_4_] and [EMIM][FAP].

### Vapor Pressure Measurement

In this experiment, a 1000 mL volumetric flask was sealed with an airtight two-hole rubber stopper. One of the holes in the stopper was connected to a manometer (Fisherbrand) and the other, to a Luer-Lock port with a stopcock for introducing liquid to the flask. The empty flask was calibrated against ambient atmospheric pressure. To measure vapor pressure, 1–2 mL of the desired liquid was squirted into the flask with a syringe through the Luer-Lock port. The vapor pressure of the liquid was measured after allowing the liquid to equilibrate for 10 min.

### Electrical Conductivity Measurement

The well-known blinking LED conductivity indicator test^29−31^ was used to segregate strong electrolytes, weak electrolytes, or nonelectrolytes. Aqueous solutions with various solutes were placed in a Chemplate for conductivity measurements. As organic solvents and ionic liquids are corrosive to Chemplate, LED conductivity indicator tests were done either in glass vials or Teflon containers. To measure the electrical conductivity of a given ionic liquid, a conductivity meter (Jenway Inc.) with a microvolume conductivity probe was used. The conductivity meter was calibrated against a standard aqueous solution, but unlike the general procedure of washing the probe with deionized water, the probe was washed with the investigated ionic liquid at least three times before dipping the probe into a given ionic liquid.

## Hazard

Ionic liquids are nonvolatile but can be corrosive and toxic to marine life with unknown effects on human health.^32^ So, students should be advised to wear goggles, laboratory coats, and gloves. Though most ionic liquids are recovered after the end of the laboratory session, any wastage should be kept separate and not be mixed with other molecular solvents for disposal. When using benzene or volatiles, students should be advised to wear appropriate masks in addition to other personal protective equipment to avoid detrimental effects, and they should preferably run the experiments in fume hoods.

## Results and Discussion

**Figure 2:**
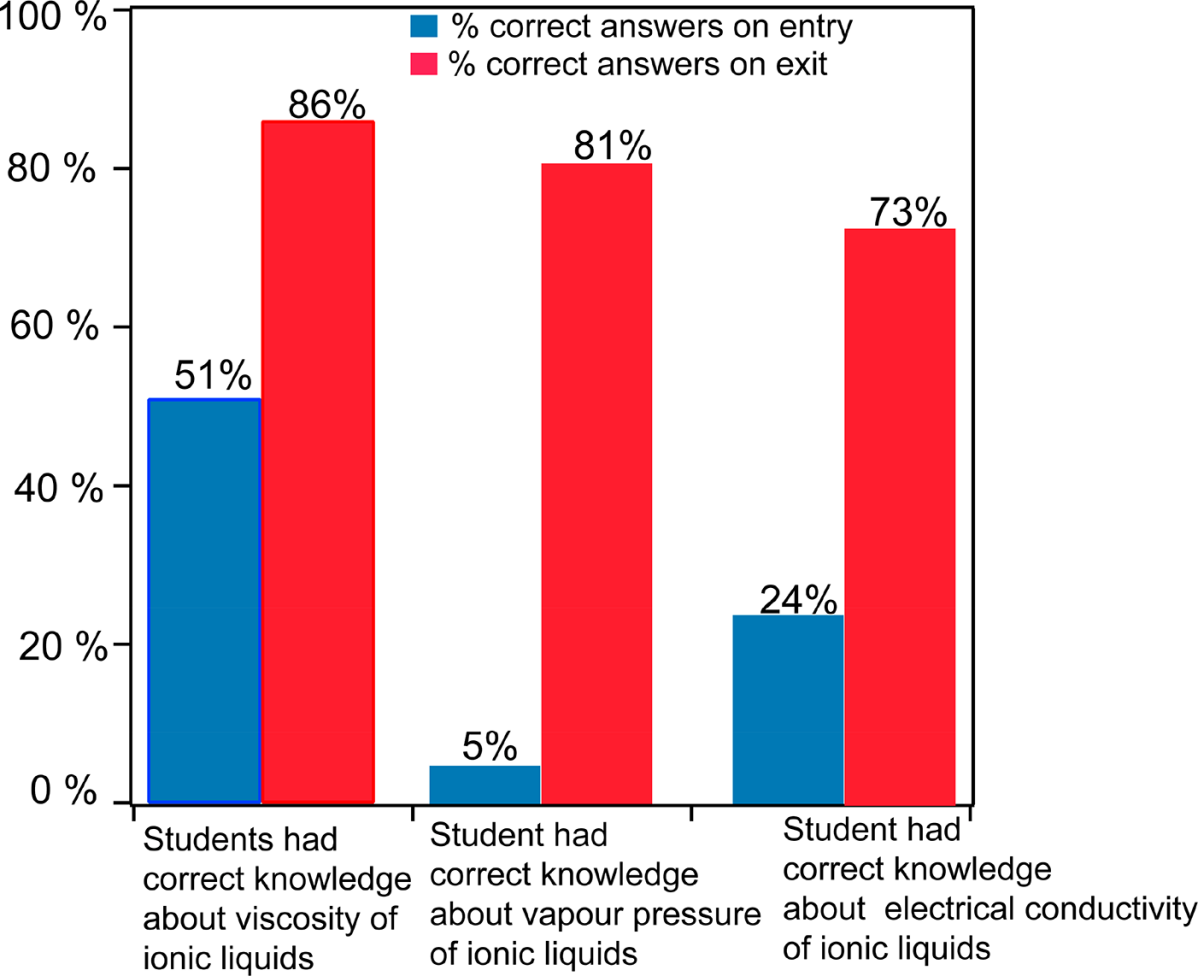
Correct responses to multiple-choice questions on the topic of viscosity, vapor pressure, and electrical conductivity before and after POGIL-inspired experiments.

Over 70% of students responded that they were not aware of ionic liquids in the entry assessment quiz. The students who were aware of ionic liquids had direct or indirect interactions with the author’s ionic liquid research through posters and research presentations on campus. It is also assumed that due to the small size of the university there was peer-to-peer dissemination of information on the research. As anticipated, more than 70% of students (Figure 2) were wrong in their assumption of the nature of electric conductivity and vapor pressure of ionic liquids in their entry assessment. Surprisingly, over 50% of students had the correct answer for the viscosity trend in ionic liquids. Follow-up in-class discussion suggested that a majority of the students did not have valid scientific reasonings supporting their choices. Overall, the entry assessment suggested that students lacked content knowledge on ionic liquids, and thus, the succeeding laboratory workflow, which began with a lecture, was pertinent to student’s academic growth.

**Figure 3:**
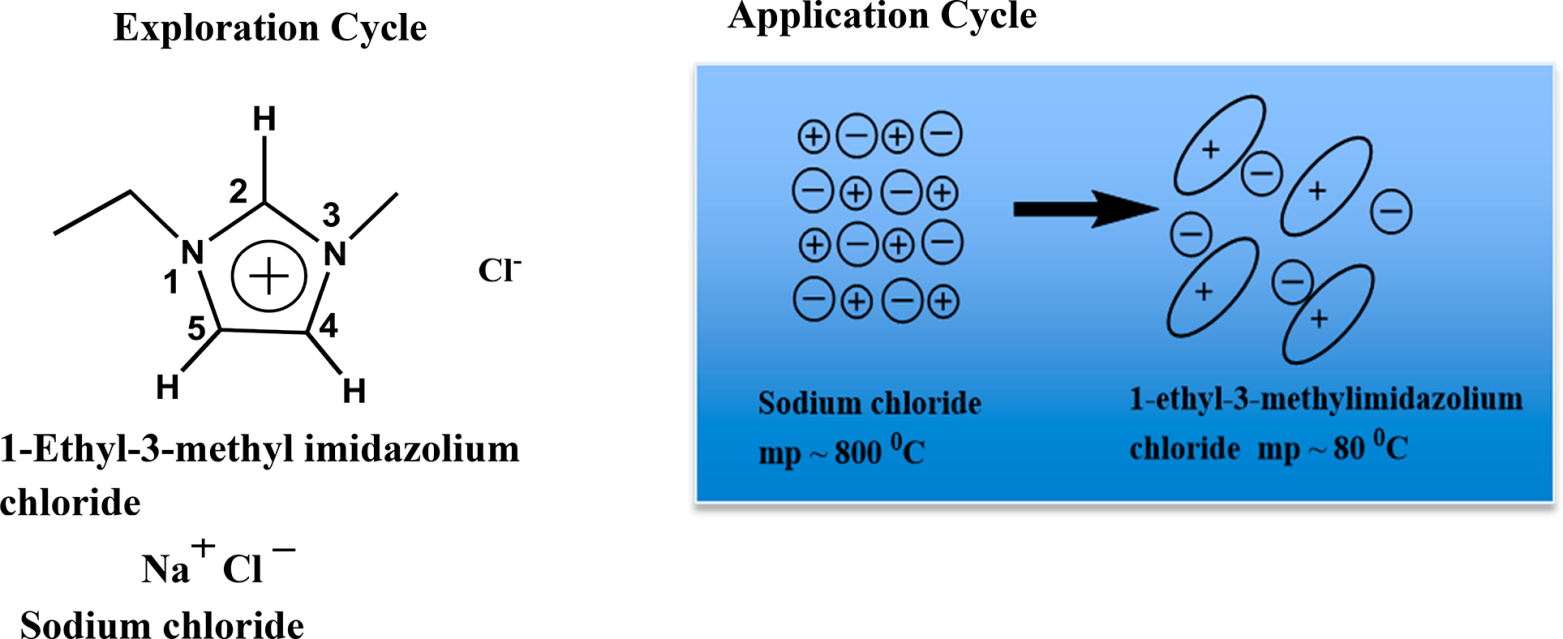
Representative structures presented in exploration and application cycle to guide students to think critically on structures of ionic crystals and ionic liquids, in terms of their relationship to melting point.

The topic of ionic liquids is generally not taught in our primarily undergraduate university. We expect a similar state in other primarily undergraduate universities. So, it is important that students are provided background knowledge on ionic liquids through a traditional lecture at the beginning of the laboratory session, followed by inquiry-based activities. Many iterations show that it is best to use the melting point as an example for inquiry-based activities to develop students’ understanding of the properties of these unique liquids. Our experience shows that guiding students in the exploration cycle (Figure 3, left), in comparing sodium chloride with the ionic liquid,1-ethyl-3-methyl imidazolium chloride ([EMIM][Cl]), reactivates concepts including but not limited to charges, size, intermolecular forces, and symmetry among students. Such guided exploration can be enhanced with a traditional lecture with a PowerPoint presentation of the charge distribution of cations and anions of ionic liquids using computational molecular electrostatic potentials. Though electrostatic potentials can be used in the application cycle to probe students’ understanding, we suggest using cartoons as shown in Figure 3 (right) and guiding discussions focusing on weakened Coulombic interaction among ions and lower lattice energy in ionic liquids due to bulky, asymmetric, and charge-delocalization in ions as origins of lower melting point. The instructor should also reflect on other forces, such as van der Waals force, and hydrogen bonding, besides the dominant Coulombic interaction in ionic liquids, and discuss how intermolecular forces are similar or dissimilar to molecular solvents. The success of this strategy is evident from the exit survey (Figure 2), where 83% of the students correctly identified the structure–function relationship regarding the melting point of ionic liquids.

Our experience shows that viscosity measurements, particularly the viscosity race experiment, is a simple but engaging way to introduce ionic liquids to students. Instead of cardboard or paper, we used an FEP film for the experiment, as it is chemically resistant. Though benzene is used as a solvent among others in the experiment, instructors should consider other similar solvents such as toluene or xylene. Using the premise that slower liquids in the race had higher viscosity, the students were able to self-realize that the viscosity of ionic liquid was higher compared to common solvents and comparable to motor oil or glycerol (Table 1). A traditional style lecture that followed the exploratory cycle emphasized the absolute viscosity value of different liquids ranging from water to honey and gave students a perspective on the possible range of viscosity of imidazolium-based ionic liquids. However, an open-ended question on the viscosity range of imidazolium-based ionic liquids yielded mixed results. The use of the Cannon-Fenske routine viscometer gave students a better idea of the dynamic viscosity of a given ionic liquid in the application cycle.

**Table 1:** Viscosity Experiments^a^

Exploration Cycle	Application Cycle	
Viscosity Race Experiment	Canon-Fenske Viscometer	
Acetone (0.36 cP), benzene (0.59 cP), water (0.89), DMSO (2.0 cP), motor oil-grade 5W-30 (54 cP), glycerol (954 cP)	[EMIM][FAP]	51 ± 6 cP (60.53 cP)
Ionic liquids: [EMIM][BF_4_], [EMIM][Tf_2_N], [EMIM][EtSO_4_], [EMIM][FAP]	[EMIM][EtSO_4_]	90 ± 3 cP (92.2 cP)

a The numbers in brackets are taken from literature values^33,34^ or from the PubChem database.

The Cannon-Fenske routine viscometer requires relatively large volumes of investigated liquid. Here, we use two ionic liquids, [EMIM][EtSO_4_] and [EMIM][FAP], for measurements as they are relatively inexpensive compared to other ionic liquids. In-class experimental values for [EMIM][EtSO_4_] and [EMIM][FAP] (Table 1) were approximately 90 and 51 cP, which were close to literature-reported values.^33,34^ As mentioned in our methodology, our goal was to give students qualitative knowledge about ionic liquids, in this case, viscosity. Due to limitations with the class setup, we only experimented with two liquids, which might give a false impression to the students that ionic liquids have a viscosity of ∼100 cP. To avoid such misconception, we suggest the instructor tabulate the viscosities of various ionic liquids in the postapplication cycle discussion. It is also pertinent that follow-up discussion should include the possible origins of viscosity in ionic liquids with comments on Coulombic compaction and charge network interactions.^35^

Ionic liquids have no or negligible vapor pressures. Vapor pressure is thus a striking property of these solvents. Over 90% of students answered the query on vapor pressure incorrectly in their entry assessment (Figure 2). The overwhelmingly wrong response is possibly due to the preconceived notion that all liquids have vapor pressure. In the exploration cycle, students were asked to measure the vapor pressure of two common solvents, hexane and heptane. Vapor pressure was determined by introducing the liquid in a sealed flask as described in the methodology. The results are presented in Table 2. Similar experiments were done earlier in the semester, and thus, students could quickly recall the concept of vapor pressure and the experimental technique. As vapor pressure is unique for ionic liquids, the students were given a short lecture revisiting the concepts taught at the beginning of the class on the forces that dictate ionic liquid properties. At the end of the lecture, the students were again asked about the possible range of vapor pressures of ionic liquids. Verbal assessment showed that the majority of students were wrong in predicting the vapor pressure of the ionic media. As such, the students were asked to repeat the same experiment as above except with instructor-provided ionic liquids. As the vapor pressure of the ionic liquid was not measurable in our experimental setup, the results elicited excitement among students. We suggest that the postapplication cycle lecture should reflect on Coulomb interactions that exist between the ions and the long-range of the interactions in the context of this unique property of vapor pressure of ionic liquids.^36^

**Table 2:** Vapor Pressure Experiments^a^

Exploration Cycle		Application Cycle	
	Vapor Pressure		Vapor Pressure
Hexane	115 ± 10 mmHg (120 mmHg)	[EMIM][FAP]	None Detected
Heptane	35 ± 8 mmHg (35.5 mmHg)	[EMIM][EtSO_4_]	None Detected

a The numbers in brackets are literature values collected from the NIST Web site at 20 °C.

Ionic liquids conduct electricity as they have ions. As is evident from the entrance ticket knowledge test, many junior or senior-level students have content knowledge on the conduction property of ionic material. More than 20% of the students correctly predicted that the ionic liquids would conduct electricity. However, the students lacked the perspective of how they should compare the conductivity of the liquid ionic media presented to them with that of aqueous electrolytes. Using the inquiry-based strategy inspired by POGIL, the students were asked to compare the relative electrical conductivity of several liquids using a blinking LED conductivity indicator. Two weak electrolytes (0.1 M NH_3_ and 0.1 M CH_3_COOH), two strong electrolytes (0.1 M KI and 0.1 M HCl), and two nonelectrolytes (distilled water, 0.1 M glucose) were placed in a Chemplate and tested with the LED indicator. In weak electrolytes, the LED light glowed faintly and did not blink, whereas the light was bright and blinked for strong electrolytes. In a parallel experiment, organic solvents benzene, acetone, hexane, and heptane were also tested using the indicator. We observed that the students were able to access their prior knowledge and quickly sorted the given liquids into three categories: weak, strong, or nonelectrolyte (Table 3). Once students’ prior knowledge was activated, they were asked to predict the nature of the pure ionic liquids in terms of weak, strong, or nonelectrolyte. Verbal assessment showed mixed results. Many students had the misconception that ionic liquids would behave like strong electrolytes. In the application cycle, the LED indicator test (Table 3) showed that the investigated ionic liquids were weak electrolytes, which is in line with the literature.^37^

**Table 3:** LED Indicator Test

Exploration Cycle			Application Cycle
Strong Electrolyte	Weak Electrolyte	Nonelectrolyte	
0.1 M KCl	0.1 M NH_3_	Distilled Water	[EMIM][BF_4_], [EMIM][Tf_2_N]
0.1 M HCl	0.1 M CH_3_COOH	Benzene, Hexane, Acetone, Heptane	[EMIM][EtSO_4_], [EMIM][FAP]
			Inference: Weak Electrolyte

Among the tested ionic liquids, [EMIM][FAP] is the best for repeated measurements in our classroom settings. Ionic liquids like [EMIM][EtSO_4_] tend to absorb water and can give false results. It is therefore necessary to dry the liquids (See the Supporting Information). We suggest that the postapplication cycle discussion/lecture should involve the instructor guiding students in differentiating aqueous solutions and pure ionic liquids in the context of the experiments. Our experience also indicates that adding an additional experiment where the students measure the electrical conductivity of given ionic liquids with a conductivity meter provides students with better insights into ionic liquids. Unlike a conventional protocol where the probing electrode is usually washed with deionized water, we recommend washing the cell with the targeted ionic liquid. This step is necessary as water contamination on the probe gives higher conductivity readings. Our in-class experiments with [EMIM][BF_4_], [EMIM][Tf_2_N], [EMIM][EtSO_4_], and [EMIM][FAP] showed that conductivity of the ionic liquids was between 1 and 15 mS/cm. The conductivity measurements were qualitative, with approximately 20% error between the measured and literature-reported values of the individual liquids.^34,38^ Typically, the conductivity of imidazolium-based ionic liquids is in the range of 1–10 mS/cm, and thus, the results were within a reasonable range. It is important that the instructor reflect with the students on the paradoxical physical mechanism of electrical conductivity in ionic liquids.^39^ Our experience suggests that, without such reflection, a student assumes ion motion in ionic liquid similar to aqueous electrolyte solutions.

Ionic liquids are nonhomogeneous and nonaqueous liquids that are increasingly becoming industrially relevant.^40^ However, it is unlikely that undergraduate students will encounter these liquids in their current undergraduate curriculum, but there are initiatives in this direction.^41^ As ionic liquids are entirely made of ions, many of their properties are unlike traditional solvents. We launched an inquiry-based laboratory module in our upper-division physical chemistry laboratory course to facilitate students’ knowledge in this area. The exercise allowed students to gain hands-on experience with ionic liquids and knowledge of the structure, intermolecular forces, and their relationship to the observed properties of ionic liquids. Analysis of the summative assessment (Figure 2) in the final examination was a clear indicator of the success of our strategy. The exit assessment showed a jump from 51% to 86% of correct responses from students on the viscosity property of ionic liquids when compared to entry evaluation. Similarly, the exit assessment showed that ∼83% of students correctly identified the nature of vapor pressure in ionic liquid as opposed to ∼5% in the entry ticket. Similarly, ∼73% of students were able to correctly determine the nature of conductivity of ionic liquid in their final assessment, indicating the success of our strategy.

## Conclusion

Ionic liquids are nonaqueous solvents entirely made of ions, which are likely to be prevalent in laboratory and industrial settings in the near future. Thus, it is necessary that undergraduate students are exposed to these nonaqueous solvents. Such exposure is particularly relevant for underrepresented undergraduate students in minority-serving institutes like ours, as they traditionally lag behind in the rapidly changing needs of the scientific workforce. To this end, we implemented an inquiry-based laboratory module with elements from POGIL combined with traditional lectures to introduce ionic liquids. The goal was not only to acclimatize students to ionic liquids but also to reflect on the investigated property’s trends and origins, which can be strikingly different from common solvents. Exit assessment showed that a majority of the students were able to grasp the concepts. Future experiments will be built on current experiences, particularly on viscosity and conductivity, to show how these properties change with the nature of the cation and anion of the ionic liquids. For example, students can be guided to predict viscosity or conductivity of a binary mixture of ionic liquids based on ideal mixing. Students can use Cannon-Fenske viscometers or conductivity meters to infer whether the mixture will show ideal or nonideal behavior. A series of such experiments where the cation or anions are systematically varied in the mixture can highlight the importance of empirical trends in relation to the structure–property relationships of these liquids and the complexities associated with assigning a specific single structural factor on the observed ionic liquid property.

## Supporting Information Available

Entry and exit questionnaire (PDF; DOCX)
Instructor guide (PDF; DOCX)
Example handout of class activities (PDF; DOCX)
