# Supplementary material for: Experiences with an Inquiry-Based Ionic Liquid Module in an Undergraduate Physical Chemistry Laboratory
Source: J Chem Educ. 2024 Apr 5;101(5):2022–9. doi: 10.1021/acs.jchemed.3c00871 (PMC11097389; doi:10.1021/acs.jchemed.3c00871)
Supplement: Supplementary file 2 — ed3c00871_si_002.docx [file ed3c00871_si_002.docx]

Supporting Information

**Experiences with inquiry-based ionic liquid module in an undergraduate physical chemistry laboratory**

Kevin E. Riley and Samrat Dutta^*^

^*^Department of Chemistry, Xavier University of Louisiana, New Orleans, Louisiana, USA.

^*^email: sdutta@xula.edu

Phone: +1 504 520 5820

**Table of Contents**

S1. Entry questionnaire.

S2. Exit questionnaire.

**S1. Entry questionnaire**.

The following are the questions that were asked upon entry to the class and without any prior information of the topic.

1) Are you aware of a class of fluids called ionic liquids?

a) Yes b) No

2) Conventional ionic liquids comprise a large asymmetric organic cation and an organic or inorganic anion*.* Unlike traditional liquids like water or methanol, these liquids are entirely made of ions with strong columbic interactions between the ions. Like any other liquids, vapor pressure is an important parameter of these liquids. From your prior knowledge and the information provided, what do you predict is the vapor pressure of these liquids?

a) Ionic liquids have a high vapor pressure.

b) Ionic liquids have a low vapor pressure.

c) Ionic liquids have no or negligible vapor pressure.

3) Ionic liquids are essentially molten salt. From this information and prior knowledge, what do you think is the electrical conductivity of ionic liquids?

a) Ionic liquids have no conductivity.

b) Ionic liquids conduct but behaves like a weak electrolyte.

c) Ionic liquids conduct but behaves like a strong electrolyte.

4) Viscosity is defined as the measure of the resistance of a fluid to gradual deformation by shear or tensile stress. What do you think is the viscosity of ionic liquids?

a) Ionic liquids have a high viscosity.

b) Ionic liquids have a low viscosity.

c) Ionic liquids have no viscosity.

**S2. Exit questionnaire**.

The following are the questions that were in the final examination. The wording and the sequence are changed each semester to preserve authenticity.

1. Imidazolium-based ionic liquids have:

(a) High vapor pressure comparable to volatile organic compounds like acetone.

(b) Low vapor pressure comparable to water.

(c) No vapor pressure or negligible vapor pressure.

(d) Intermediate vapor pressure between volatile organic compounds and water

2. Imidazolium-based ionic liquids:

(e) Behaves like non-electrolytes with no conductivity.

(f) Behaves like weak electrolytes with conductivity in 1-10 mS/cm range.

(g) Behaves like strong electrolytes with conductivity in 1-10 S/cm range.

(h) Behaves like highly conductive metal with conductivity in the range of 10^4^-10^5^ S/cm.

3. Imidazolium-based ionic liquids have:

(i) A lower viscosity than water or traditional organic liquids.

(j) A higher viscosity than water or traditional organic liquids.

(k) A similar viscosity when compared to water or traditional organic liquids.

(l) No viscosity.

4. Many imidazolium-based ionic liquids are liquids at room temperature because:

(m) They have strong interionic interactions like sodium chloride.

(n) They have asymmetric charge distribution either due to the nature of the cation or
 anion or both resulting in poor packing and weaker interionic interactions.

(o) They have symmetric charge distribution as both cation or anion are small resulting in
 dense packing and weaker interionic interactions.

(p) They have only Vander Waals and hydrogen bonding and do not have ionic interactions.
