# Supplementary material for: Experiences with an Inquiry-Based Ionic Liquid Module in an Undergraduate Physical Chemistry Laboratory
Source: J Chem Educ. 2024 Apr 5;101(5):2022–9. doi: 10.1021/acs.jchemed.3c00871 (PMC11097389; doi:10.1021/acs.jchemed.3c00871)
Supplement: Supplementary file 4 — ed3c00871_si_004.docx [file ed3c00871_si_004.docx]

Instructor guide

**Experiences with inquiry-based ionic liquid module in an undergraduate physical chemistry laboratory**

Kevin E. Riley and Samrat Dutta^*^

^*^Department of Chemistry, Xavier University of Louisiana, New Orleans, Louisiana, USA.

^*^email: sdutta@xula.edu

Phone: +1 504 520 5820

**Instructor guide**

Students should work in pairs or, at most, in a group of four. The instructor should place the ionic liquids in a high vacuum and heat them to 80^0^ C for at least 2 hours before the start of the class. Most of the ionic liquids used in the experiments can be recovered and reused for at least two years without loss of function. Water contamination, if any, can be quantified using free water bands in the infrared using the “free water” bands at 3640 and 3560 cm^−1^ and should typically be less than 1000 ppm.^1^ The instructor should consider long-term storage of ionic liquids in vacuum desiccators.


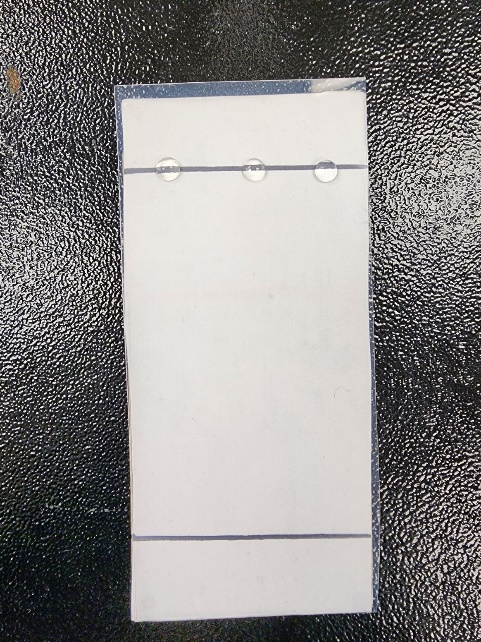


[EMIM][BF_4_]

DMSO

H_2_O

FEP Flim

20 μL Each

**Viscosity measurements**

Viscosity race measurements are meant for qualitative results and not for quantitative measurements. This experiment is designed to give students their first experience in handling ionic liquids. To this end, each group is given a homemade flat block of wood whose surface is protected with an FEP film (Club 3D) as shown in Figure S1. The block should have a start line and finish line. At the beginning of the experiment, the instructor should hand out 100 μL of an ionic liquid and two other molecular liquids (e.g., water) to each group in 0.5 ml Eppendorf tubes. Different groups should be given different combinations of liquids. The choice of molecular solvents should be based on the vapor pressure of the liquid. At least one group should be given high-vapor pressure molecular solvents like acetone.

**Figure S1:** 20 μL drops of liquids are placed equidistant for viscosity race.

With a fully adjustable, air-displacement pipette (e.g., Pipetteman, Gilson Inc) the students should be instructed to draw 20 μL of the given liquids. Students must be consistent in this part of the exercise. The 20 μL drops of one ionic liquid and two molecular solvents should be placed equidistant below the start line horizontally on the wooden block. The timer should start the moment the block is raised to ~45^0^. Alternatively, the experiment can be done sequentially with one liquid at a time from the wooden block already at ~45^0^. The timing of the same liquid reaching the finish line can be different for different groups depending on the block dimension, but the relative timing should follow the same pattern. Viscous liquids flow slower. As very low volumes of liquids are used for this exercise, paper towels can be used to collect the runoff and clean the FEP surface. The paper towels should be discarded in a labeled container. The remaining ionic liquid in the Eppendorf tube from each group should be recycled.


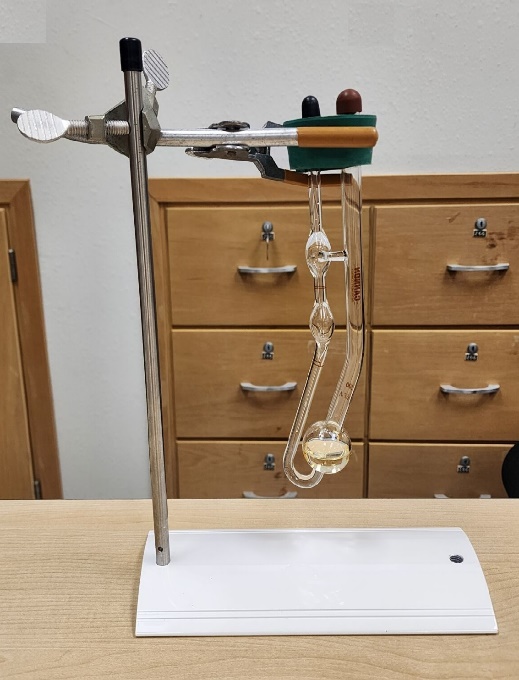


**Figure S2:** Cannon-Fenske routine viscometer with ionic liquid. The liquid can be stored in the apparatus and reused after drying.

[EMIM][ETSO_4_]

We suggest the use of volatile liquids in the above experiment to serve as a talking point when exploring vapor pressure. Moreover, using volatile liquids in the experimental workflow, which includes activities such as pipetting, setting up the drops, observing flow characteristics, and other aspects, allows the students to self-discover differences between volatile, less viscous liquids and non-volatile viscous ionic liquids.

Cannon-Fenske routine viscometer measurements require ~6-8 mL each of the ionic liquids, [EMIM][EtSO_4_] and [EMIM][FAP]. The ionic liquid is drawn into the upper bulb by suction and then allowed to flow down through the capillary into the lower bulb between two marks. Kinematic viscosity is determined from the time taken for the ionic liquid to pass between these marks. The dynamic viscosity of the ionic liquid is calculated from the kinematic viscosity. After the experiment, the ionic liquid is stored in the viscometer by capping the two-open ends (See Figure S2). For reuse, the entire apparatus should be inserted in a vacuum-drying oven and heated for several hours at 80 ^0^C. [EMIM][EtSO_4_] requires a longer time to remove absorbed water than [EMIM][FAP].


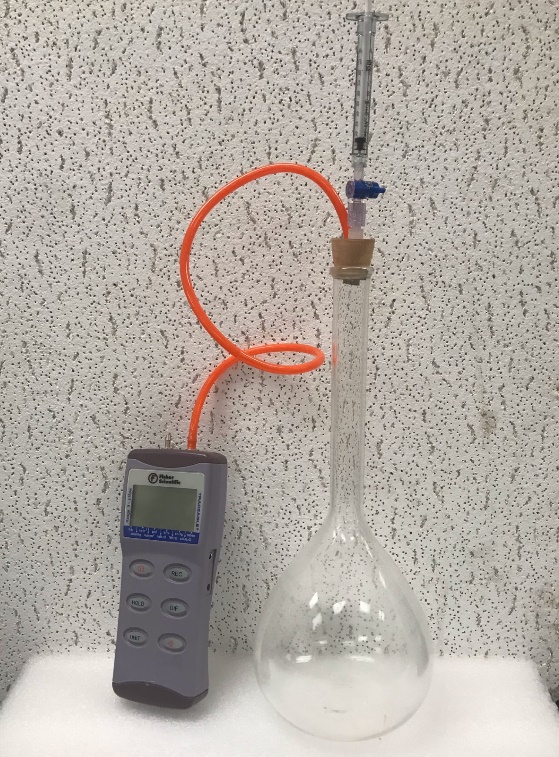


Injection

Port

**Vapor pressure measurements**


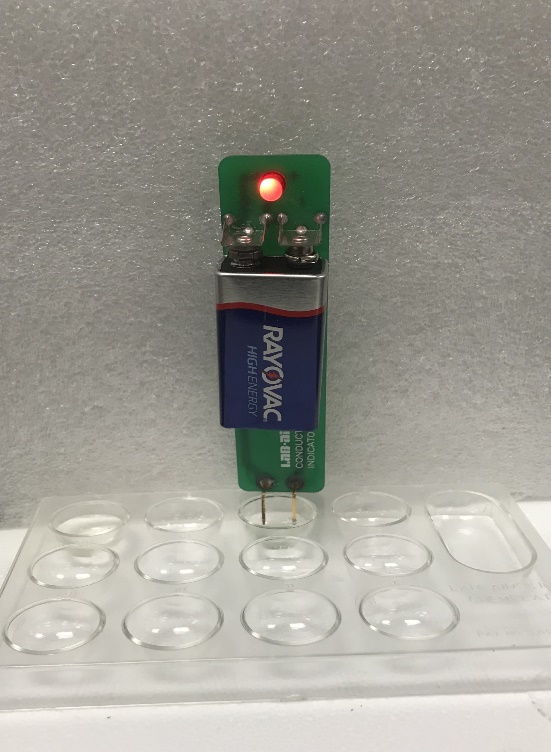
A 1000 mL oven-dried empty volumetric flask is capped with a rubber stopper with two holes. One end of a hard-wall tubing is inserted into one of the holes of the rubber stopper, with the other end of the tubing attached to one of the barb ports of a digital differential manometer (Traceable®, Cole-Palmer Inc). A plastic Luer-Lock connector with a tapered, ribbed tip (Stopper Stem, Vernier) is inserted into the rubber stopper's other hole (injection port). A two-way value (Plastic 2-Way Valve, Vernier) is attached to this connector, which is, in turn, connected to a Luer-Lock syringe. The arrangement of the set-up is shown in Figure S3. The two-way valve at the injection port should be in a closed position at the start of the experiment, with the manometer set to zero differential pressure with the atmosphere. Approximately 1 mL of the investigated liquid should be taken with a plastic syringe, and the same should be delivered into the volumetric flask by opening the two-way valve with the syringe. The valve should be immediately closed after injecting the liquid. Manometer readings should be taken after 10 minutes. The injected liquid must be noticeable at the bottom of the flask. Ionic liquids can be recovered by washing the vessel with methylene chloride and removing the volatile solvent by rotary evaporation.

**Figure S3:** Vapor pressures set-up for both molecular solvents and ionic liquids.

**Electrical conductivity measurement**

As shown in Fig S4, wells of a spot plate (Chemplate^TM^) should be filled with drops of liquid enough to submerge the electrodes of the LED blinking indicator (Lab-Aids^TM^) to identify strong electrolytes, weak electrolytes, or non-electrolytes. The electrodes of LED indicator must be washed with deionized water and dried before testing another liquid in the spot plate. Note that all the liquids for this exercise should be aqueous solutions of the same concentration.

**Figure S4:** LED blinking indicator dipped in 0.1 M HCl (Strong Electrolyte) in spot plate shows bright blinking light.

To test conductivity of organic solvents, it is recommended that the instructor uses 20 mL glass vials that are fully filled with the desired liquid. It is recommended that this experiment be done in a fume hood. We suggest inserting the vials in an aluminum block with holes or in plastic inserts, as shown in Figure S5, to prevent accidental spillage. The student should sequentially perform the experiment, opening a selected vial, inserting the electrode, recording the result, closing the vial, and then proceeding to the next second vial. There should be no response from the indicator. To avoid cross-contamination, the electrodes of the indicator should be washed with water and dried between two organic solvents.


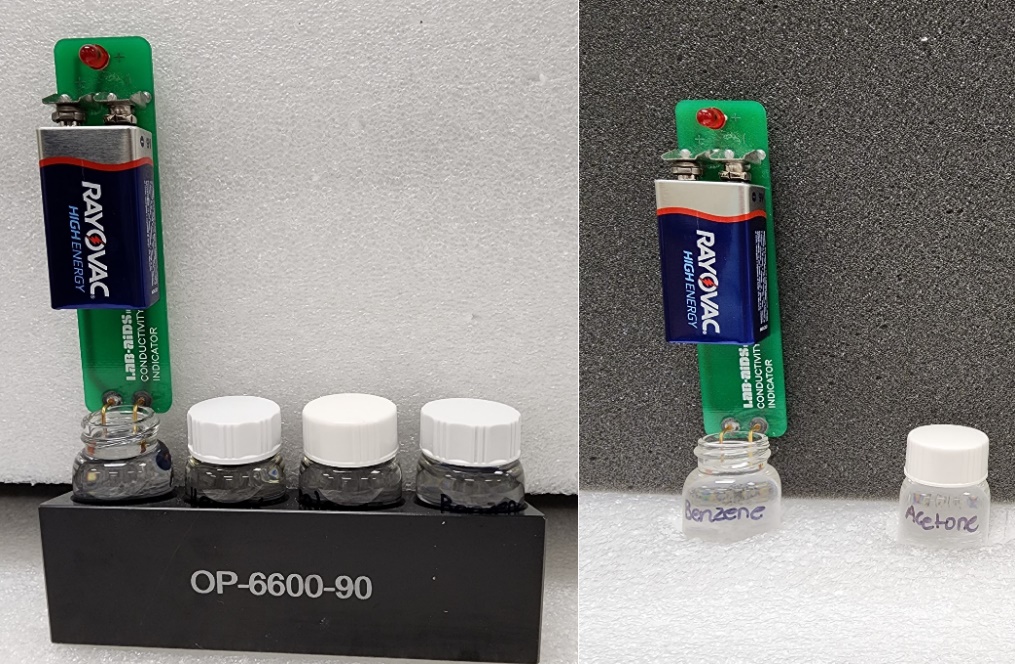


Benzene

Acetone

Hexane

Heptane

**Figure S5:** Two arrangements to study conductivity of organic solvents considering safety.


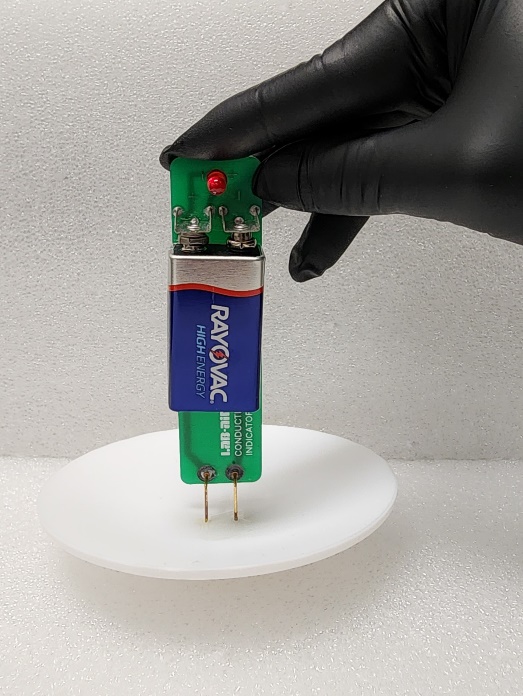
Conductivity tests of ionic liquids should be done in Teflon-coated wells such as PTFE-watch glass (PTFE Watch Glasses, Fisherbrand™), as shown in Figure S6. 150-200 μL of the selected ionic liquid is enough to have good electrical contact with the LED indicator electrodes. Hydrophobic ionic liquids such as [EMIM][FAP] gives the best result. It is to be noted that all dry ionic liquids show the same result. The LED blinking indicator should show a dim glow, as shown in Figure S6. Students who had earlier performed conductivity tests on strong, weak, or non-electrolytes can unambiguously infer that ionic liquids act as weak electrolytes. The ionic liquid can be recovered after testing.

A benchtop conductivity meter (Jenway, Cole-Parmer Inc.) with a micro-volume conductivity probe with a 4 mm shaft diameter was used to measure the conductivity of ionic liquids (Figure S7). The equipment should be calibrated with a standard conductivity solution before the start of the experiment. The probe should be kept in deionized water in between measurements. For measurements, the probe should be sequentially washed in 0.5 mL Eppendorf tube(s) containing approximately 200 μL of the ionic liquid whose conductivity is to be tested. 200 μL is sufficient to submerge the probe head electrodes completely in the liquid. Washing is accomplished by dipping the electrode for a few seconds and gently tapping the excess liquid in the tube before moving to the next Eppendorf tube (See below). It is recommended to have at least 3-4 such tubes with the ionic liquid to be tested for washing. To record the conductivity, the probe is inserted into a labeled 0.5 mL Eppendorf tube (yellow tube in Fig. S7) with the ionic and allowed to stabilize. The reading is taken 1-2 minutes after stabilization. As before, all the ionic liquids can be combined after the experiment. Heating under a vacuum ensures the recovery of the original liquid.

**Figure S6:** LED blinking indicator dipped in [EMIM][FAP] on a PTFE-watch glass shows dim illumination unambiguously showing that it is a weak electrolyte.


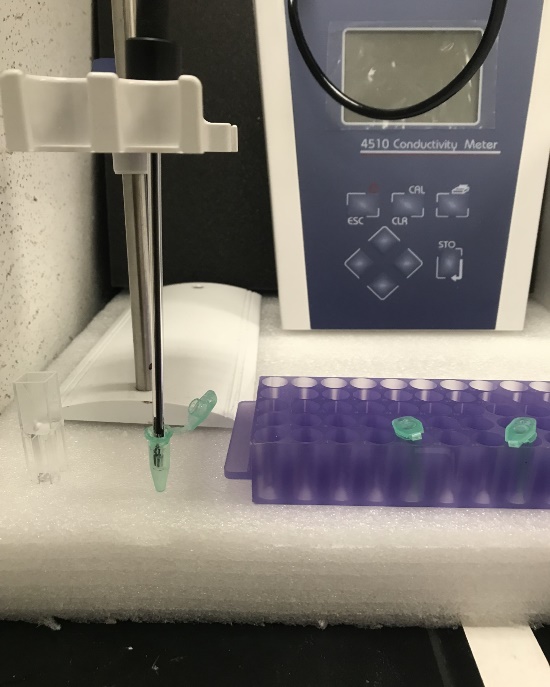

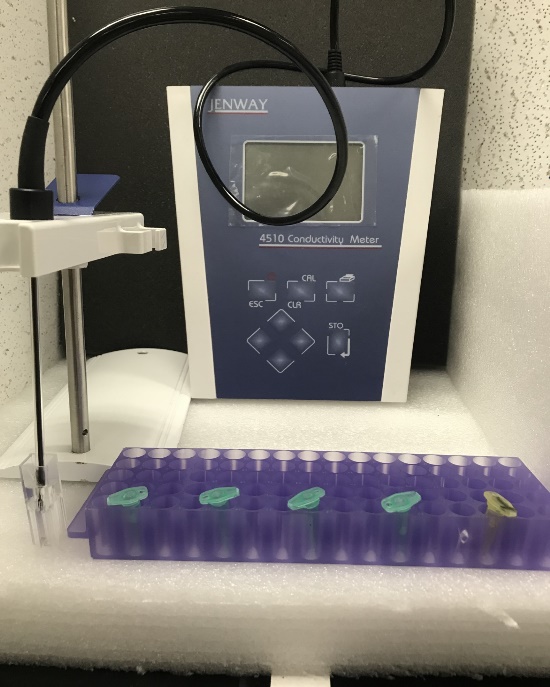


Ionic Liquid Wash Stations

**Figure S7:** (Left) The probe is first calibrated and then dipped in ionic liquid (to be tested) wash stations sequentially (right). The yellow Eppendorf tube is where the final measurement is performed.

Alternatively, the experiments can be done in vials 20 mL glass scintillation vials using any standard conductivity meter with conductivity electrode capable of measuring conductivity in the range of 1 μS/cm to 100 mS/cm. However, the method requires more ionic liquid for measurement and needs more effort to recover the ionic liquids.

**Reference**

1. Cammarata, L.; Kazarian, S. G.; Salter, P. A. ; Welton, T. Molecular States of Water in Room Temperature Ionic Liquids *Phys. Chem. Chem. Phys.,* **2001**, 3, 5192-5200.
