# Supplementary material for: Experiences with an Inquiry-Based Ionic Liquid Module in an Undergraduate Physical Chemistry Laboratory
Source: J Chem Educ. 2024 Apr 5;101(5):2022–9. doi: 10.1021/acs.jchemed.3c00871 (PMC11097389; doi:10.1021/acs.jchemed.3c00871)
Supplement: Supplementary file 5 — ed3c00871_si_005.pdf [file ed3c00871_si_005.pdf]

Handout

Experiences with inquiry-based ionic liquid module in an undergraduate physical chemistry laboratory

Kevin E. Riley and Samrat Dutta*
**Department of Chemistry, Xavier University of Louisiana, New Orleans, Louisiana, USA.*
*email: sdutta@xula.edu
Phone: +1 504 520 5820

Example handout for the learning and exploration cycle.
Most of the handouts were prompts to recollect previous knowledge aligned to Xavier University of Louisiana academic knowledge of the student.

Electric Conductivity of Liquids (Learning Cycle)
Conductivity is a measurement of the ability of fluid to transfer an electrical current. We used a simple conductivity meter before in the GenChem 1011L lab using an LED light bulb and a battery to compare the conductivity of polar, slightly polar, and nonpolar liquids.

i. *Nonconductor = No light = Nonelectrolyte*
ii. *Poor conductor = Dim light and not blinking= Weak Electrolyte*
iii. *Good conductor = Bright light and Blinking = Strong Electrolyte*

Definition:

A strong electrolyte has many ions (or completely dissociates) when dissolved in water.

A weak electrolyte has few ions (or do not completely dissociate) when dissolved in water.

Non-electrolytes do not dissociate into ions when dissolved in water.

Discuss in your group, using the above scale, the predicted LED response and your inference whether the liquid(s) given below is weak, strong, or non-electrolyte.

a) Deionized water b) 0.1 M Ammonia Solution c) 0.1 M HCl Solution

Experimental part: This part is similar to your earlier experiment in GenChem 1011L laboratory. Briefly add a few drops of test liquids given by the instructor on a clean and dry well on the Chemplate™. There should be enough liquid in the well so that the LED conductivity apparatus electrodes can be immersed into the liquid. Before testing, ensure that the electrodes are clean. The electrodes can be cleaned by immersing the electrodes in deionized water.

Record the liquid you are testing in the table below. Your group leader may be asked to fill the same in the class blackboard.

Liquids	LED response	Inference
A		
B		
C		
D		
